# Neurocognition and Subjective Experience Following Acute Doses of the Synthetic Cannabinoid JWH-018: Responders Versus Nonresponders

**DOI:** 10.1089/can.2018.0047

**Published:** 2019-03-13

**Authors:** Eef L. Theunissen, Nadia R.P.W. Hutten, Natasha L. Mason, Stefan W. Toennes, Kim P.C. Kuypers, Johannes G. Ramaekers

**Affiliations:** ^1^ Department of Neuropsychology and Psychopharmacology, Faculty of Psychology and Neuroscience, Maastricht University, Maastricht, The Netherlands.; ^2^ Department of Forensic Toxicology, Institute of Legal Medicine, Goethe University of Frankfurt, Frankfurt, Germany.

**Keywords:** cognition, impairment, JWH-018, safety, subjective experience, synthetic cannabinoid

## Abstract

**Introduction:** Synthetic cannabinoid mixtures have been easily accessible for years, leading to the belief that these products were natural and harmless, which contributed to their popularity. Nevertheless, there are many reports of users ending up in hospital due to severe side effects such as tachycardia, aggression, and psychosis. Controlled studies on the effects of synthetic cannabinoids on human performance are lacking. In the present study, we assessed the safety pharmacology of the synthetic cannabinoid JWH-018 after acute administration.

**Methods:** Seventeen healthy cannabis-experienced participants took part in this placebo-controlled, crossover study. Participants inhaled the vapor of JWH-018 (doses ranged between 2 and 6.2 mg) and were subsequently monitored for 12 h, during which vital signs, cognitive performance, and subjective experience were measured. Subjective high scores showed that there is a large variability in the subjective experience of participants. Therefore, a mixed analysis of variance, with “Responder” (i.e., subjective high score >2) as a between-subjects factor and “Drug” as a within-subjects factor (placebo and JWH-018), was used.

**Results:** Serum concentrations of JWH-018 were significantly higher in the responders. Overall, JWH-018 increased heart rate within the first hour and significantly impaired critical tracking and memory performance. Responders to JWH-018 performed more poorly in tests measuring reaction time and showed increased levels of confusion, amnesia, dissociation, derealization, and depersonalization and increased drug liking after JWH-018.

**Conclusion:** JWH-018 administration produced large variability in drug concentrations and subjective experience. Fluctuations in drug delivery probably contributed to the variation in response. JWH-018's impairing effects on cognition and subjective measures were mainly demonstrated in participants who experienced a subjective intoxication of the drug. Lack of control over drug delivery may increase the risk of overdosing in synthetic cannabinoid users.

## Introduction

During the last 10 years, there has been a steep increase in the number, type, and availability of novel psychoactive substances (NPS) worldwide. A novel psychoactive substance is defined as “a new narcotic or psychotropic drug, in pure form or in preparation, that is not controlled by the United Nations drug conventions, but which may pose a public health threat comparable with that posed by substances listed in these conventions.”^1^ Smoking mixtures that contain synthetic cannabinoids constitute one of the largest substance groups within NPS and have become a popular alternative for cannabis. Spice is one of the earliest blends of herbs being sold as an alternative for cannabis, but hundreds of different brands have come onto the market since then, with names such as K2, Black Mamba, or Yucatan Fire.^2^

Natural cannabis has been used recreationally for centuries and is still one of the most widely used drugs.^3^ The effects of cannabis on psychological and behavioral measures have been investigated in numerous experimental, placebo-controlled studies, giving us valuable information on the risk profile of this drug. These studies have shown that the effects of cannabis on performance are dependent on factors such as dose, amount of Δ9-tetrahydrocannabinol (THC, the psychoactive component in cannabis), previous experience with the drug, and time of testing after administration.^4–7^ In recreational users, THC in doses between 40 and 500 μg THC/kg body weight generally causes an acute, dose-related impairment not only of cognitive functions such as memory, attention, and reaction time (RT) but also of motor performance and actual driving.^6,8–14^ These impairments were shown to emerge at serum THC concentrations as low as 2–5 ng/mL, and were maximal during the first hour after smoking and declined rapidly thereafter.^4,15^ Unfortunately, comparable studies with NPS, such as synthetic cannabinoids, in humans are almost nonexisting. Consequently, reliable and well-validated information on individual health risks is missing. Nevertheless, this is urgently needed to provide a full-scale risk assessment of NPS.^16^

Like THC, synthetic cannabinoids act on the central cannabinoid receptors but have a much higher binding affinity for both CB1 and CB2 receptors and often act as full agonists.^17^ Consequently, the effects of synthetic cannabinoids are much stronger than that of natural cannabis, and the risk for overdosing is considerably higher. Case reports and hospital admission reports show that synthetic cannabinoids can produce a multitude of effects such as tachycardia, hallucination, seizures, anxiety, panic attacks, and acute psychosis.^17–25^

In 2008, the synthetic cannabinoid JWH-018 was identified as the active ingredient of spice.^26^ JWH-018 belongs to the aminoalkyl indole class and produces cannabis-like effects when smoked.^24^ It acts as a full CB1 agonist resulting in a strong inhibition of gamma-aminobutyric acid (GABA) neurotransmission, which can induce seizures and convulsions and therefore could lead to potentially life-threatening conditions.^27^ Previously, two self-experiments reported typical cannabis-like effects soon after administrating JWH-018 in doses up to 4.3 mg.^18,28^

Recently, we conducted a pilot study in six healthy cannabis-experienced volunteers.^29^ As JWH-018 is four to five times as potent as THC,^30^ we administered single doses of 2 and 3 mg JWH-018. It was expected that a dose of 3 mg JWH-018 would produce pharmacological effects comparable with a dose of 15 mg of THC. The latter has been shown to produce significant behavioral effects in controlled studies while keeping adverse events to a minimum.^15^ The JWH-018 doses of 2 and 3 mg were well tolerated by participants, and there were no serious health issues reported during the study or within the 72 h after drug administration. Subjective high scores and serum drug concentrations nevertheless were generally low and not fully representative of common use. Nonetheless, signs of neurocognitive impairment and subjective feelings of high did emerge, particularly after the 2 mg dose. Although we expected that the used doses would have comparable behavioral effects as an average dose of cannabis, the demonstrated effects turned out to be less strong than expected. Therefore, we have extended this pilot study, increased the dose (75 μg/kg bodyweight), and enlarged our sample to increase the statistical power.

## Methods

The study was approved by the standing Medical Ethics Committee of Maastricht University and was carried out in compliance with the current revision of the Declaration of Helsinki (amended in 2013, Fortaleza) and the International Conference on Harmonization guidelines for Good Clinical Practice. A permit for obtaining, storing, and administering JWH-018 was obtained from the Dutch drug enforcement administration. All subjects provided written informed consent and received financial compensation for their participation.

### Participants

A total of 19 occasional users of cannabis were recruited via advertisements. Participants were screened using a locally developed health questionnaire and underwent a medical examination (including an electrocardiogram [ECG], hematology and blood chemistry, urinalysis, and drug and pregnancy screening). The following inclusion criteria applied: occasional use of cannabis (to get a coherent group with a similar history of use, participants had a minimum 1 year experience with cannabis, with a minimum and maximum use of 24 and 104 times/year); free from psychotropic medication; good physical health as determined by medical examination and laboratory analyses (hematology and blood chemistry, urinalysis); absence of any major medical, endocrine, and neurological condition; body mass index (BMI, weight/length^2^) between 18 and 28 kg/m^2^; and written informed consent. Exclusion criteria were excessive drinking (>20 alcoholic consumptions/week); pregnancy or lactation or failure to use contraceptives; hypertension (diastolic >90 and systolic >140); history of psychiatric disorders; and history of drug abuse. Recent use of drugs was assessed by drug urine screens, while the Severity of Dependence Scale, Cannabis Problems Questionnaire, and a locally developed history of drug use questionnaire were screened for history of drug abuse.

One participant withdrew from the study for personal reasons not related to the study, while another participant participated in both the pilot and the added group, and therefore, data from this person from the pilot study were excluded.

### Design and treatments

The study was conducted according to a placebo-controlled, single-blinded, within-subjects design. On separate test days, each subject inhaled the vapor of a placebo or a dose of JWH-018. JWH-018 was either given in a fixed dose of 2 mg (*N*=5) or 75 μg/kg bodyweight (*N*=12; average dose was 3.95 mg). Test days were separated by a minimum washout period of 7 days to avoid cross-condition contamination.

JWH-018 powder, retrieved from THC Pharm (Germany), was mixed with a small amount (±15 mg) of Knaster Hemp (Zentauri, Germany), a herbal blend with hemp aroma (0% THC). Placebo consisted of only Knaster Hemp. Both were heated in a 10 cm glass pipe (“crack pipe”), which was replaced for every new administration. A 30 cm plastic tube was connected to the end of the pipe, while the bowl of the pipe contained the treatment. While the air holes were closed off, the bowl was heated for about 15 sec. When the vapor was formed, the air holes were opened and the subject was instructed to immediately inhale the vapor in one take via the plastic tube. Drug preparation and administration were done by a different researcher than the ones performing all other assessments.

### Procedures

Procedures and tests are described in the Supplementary Data. Safety was monitored continuously (vital signs and ECG), while laboratory analyses (hematology and blood chemistry, urinalysis), cognitive performance (digit symbol substitution [DSST], critical tracking task [CTT], divided attention task [DAT], stop signal task [SST], Tower of London [TOL], and spatial memory task [SMT]), and subjective experience (Profile of Mood States [POMS], Bowdle visual analog scales, Marijuana Craving Questionnaire [MCQ], Sensitivity to Cannabis Reinforcement Questionnaire [SCRQ], and Clinician-Administered Dissociative States Scale [CADSS]) were measured at regular times during the test days (Tables 1 and 2). Fourteen blood samples were taken during each test day for pharmacokinetic analyses.

**Table 1.** Cognitive Tests Taken During Test Days Relative to Time of Administration (T_0_)

Time (h) to T_0_	DSST	SST	CTT	TOL	DAT	SMT
Baseline	X		x			
0:15			x			
0:30						X
1:00		x			x	X
2:30			x	x	x	
4:30	X	x				
6:30			x			
8:30	X	x				
10:30			x	x	X	

DSST, digit symbol substitution test; SST, stop signal task; CTT, critical tracking task; TOL, Tower of London; DAT, divided attention task; SMT, spatial memory task.

**Table 2.** Time of Subjective Questionnaires Taken During Test Days, Relative to Time of Administration (T_0_)

Time (h) to T_0_	VAS-high	SCRQ	MCQ	POMS	CADSS	BOWDLE
Baseline	x			x		
0:05	x	x			x	
1:00	x		x	x		X
2:00	x					
3:00	x					
4:00	x				x	
5:00	x			x		X
6:00	x					
7:00	x					
8:00	x					
10:00	x	x			x	
12:00	x		x	x		X

VAS-high, visual analog scales of subjective high; SCRQ, Sensitivity to Cannabis Reinforcement Questionnaire; MCQ, Marijuana Craving Questionnaire; POMS, Profile of Mood States; CADSS, Clinician-Administered Dissociative States Scale; Bowdle, Bowdle visual analog scales.

#### Subjective high

Subjective high is self-rated on a 10 cm visual analog scale (VAS), with 0 indicating “not high at all” and 10 indicating “extremely high.” Participants indicated their subjective high at baseline, 5 min after inhalation of the drug, and subsequently at regular intervals during the test day (Table 2).

### Statistics

**FIG. 1.**
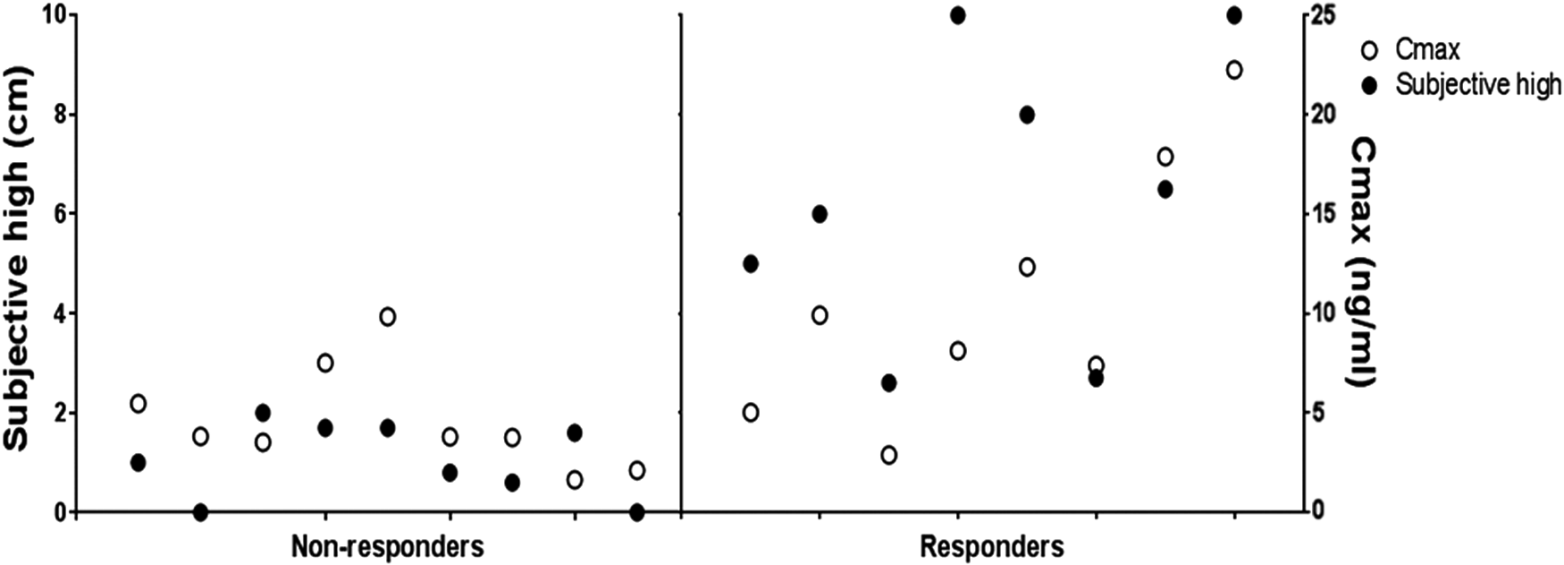
Individual's score on the subjective high visual analog scale at 1 h postadministration (black dots), and the maximum concentration of JWH-018 in serum (white dots) for responders and nonresponders.

Subjective high scores showed great variability between participants. Only eight participants reached a subjective high score larger than 2, that is, indicating subjective intoxication (Fig. 1). Therefore, we used a mixed analysis of variance, with “Responder” (i.e., subjective high score >2 [*n*=8] vs. subjective high <2 [nonresponder; *n*=9]) as a between-subjects factor and “Drug” (placebo and JWH-018) and “Time” (repetition of the test, see Tables 1 and 2) as within-subjects factors. Vital sign measurements were divided in three time intervals (i.e., within the first hour, between 75 and 360 min, and between 390 and 720 h after inhalation) and analyzed separately. A Greenhouse/Geisser correction was applied in case of violation of sphericity. One-sided testing was used, as we expected JWH-018 to cause impairment compared with placebo. A *p*-value of <0.05 was considered statistically significant. All statistical tests were conducted using IBM SPSS statistics, version 24.

## Results

Data from 17 subjects (7 males and 10 females) were analyzed. On average (standard deviation [SD], min–max), participants were 23.4 years old (3.1, 18.8–28.8), had a BMI of 22.3 (1.9, 19.5–27.2), and used cannabis for 5.7 years (3.4, 1–13), 1.4 times a week (0.6, 0.5–2.5).

Although subjects were instructed to abstain from cannabis as of 5 days before each test day, four subjects tested positive for cannabinoids at baseline. These participants had 2.1, 0.8, 0.51, and 0.4 ng/mL THC in serum, which indicates that last use of cannabis was probably a couple of hours or days before the start of the test day. THC concentrations below 2 ng/mL, however, are not associated with psychomotor impairment.^4^ This indicates that psychoactive effects of THC were negligible at the start of the test days, which is indeed confirmed by a baseline subjective high score of zero for these participants.

Due to technical malfunctioning, the SMT data from one participant were missing.

### Safety

**FIG. 2.**
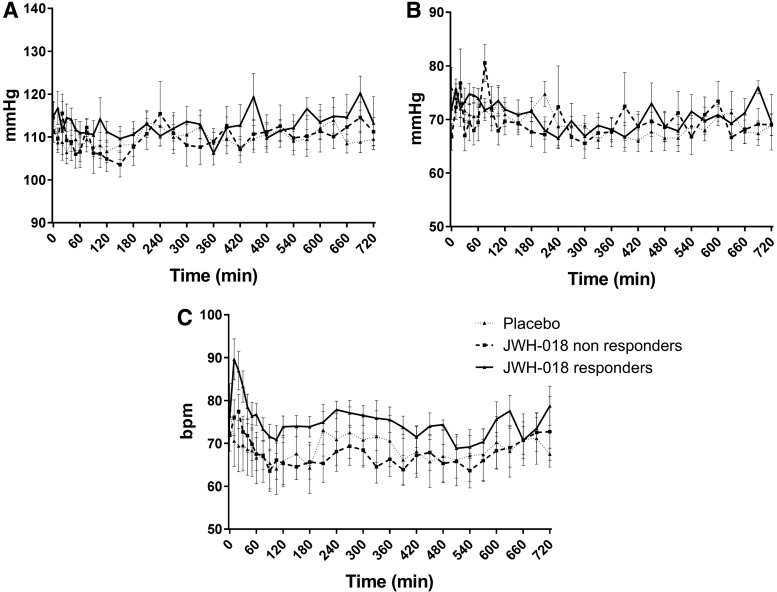
Average (±SEM) values for systolic **(A)** and diastolic blood pressure **(B)** and heart rate **(C)** for placebo and JWH-018 in responders and nonresponders. SEM, standard error of the mean.

Laboratory analyses (hematology, clinical chemistry, and urinalyses) showed no clinically relevant deviations from normal ranges. ECG patterns and vital signs measured with Dyna-Vision were also normal. Average blood pressure and heart rate (Omron measurement) are presented in Figure 2. Heart rate increased within the first hour after administration of JWH-018 (*F*_1,15_ = 13.29; *p*=0.001). Between 6 and 12 h after administration of JWH-018, systolic and diastolic blood pressure was significantly higher compared with placebo (*F*_1,15_ = 3.87; *p*=0.034; *F*_1,15_ = 4.12; *p*=0.031).

No side effects were reported during the test days, except for one participant feeling light headed during blood taking in the placebo condition. Seven participants reported side effects after the end of the test day: two participants reported headaches in the placebo condition, while one participant reported a headache after JWH-018 treatment; three participants reported low energy/tiredness after JWH-018; and one participant reported an irregular heart beat after JWH-018. This last side effect was followed up by the medical doctor who decided that it was not clinically relevant.

### Pharmacokinetics

**FIG. 3.**
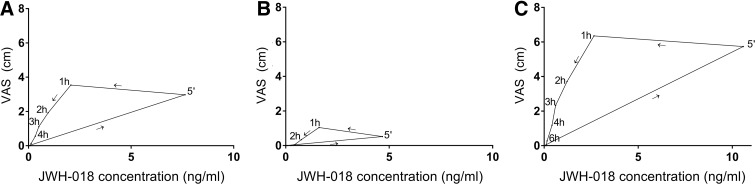
Average subjective high score plotted against average JWH-018 serum plasma concentrations over time after administration, in the total group **(A)**, the nonresponders **(B)**, and the responders **(C)**.

Maximal JWH-018 concentrations in serum differed substantially between participants, ranging from 1.65 to 22.26 ng/mL (mean=7.49; SD=5.66). Mean JWH-018 concentrations over time are given in Figure 3. The highest drug concentrations were observed within 15 min after inhalation and reached an average (±SD) of 7.63 (5.79) ng/mL in the total group, 4.67 (2.64) ng/mL in the nonresponders versus 10.59 (6.19) ng/mL in the responders. An independent *t*-test showed that this difference was significant (*t*_15_=−2.58; *p*<0.05).

The glass pipes of the 12 participants who received 75 μg/kg bodyweight of JWH-018 showed an average (min–max) residue of 57% (24–83%). For responders in this subgroup, the average residue in the pipes was 46% (24–64%), while this was 68% (44–83%) in nonresponders.

### Cognitive performance

**FIG. 4.**
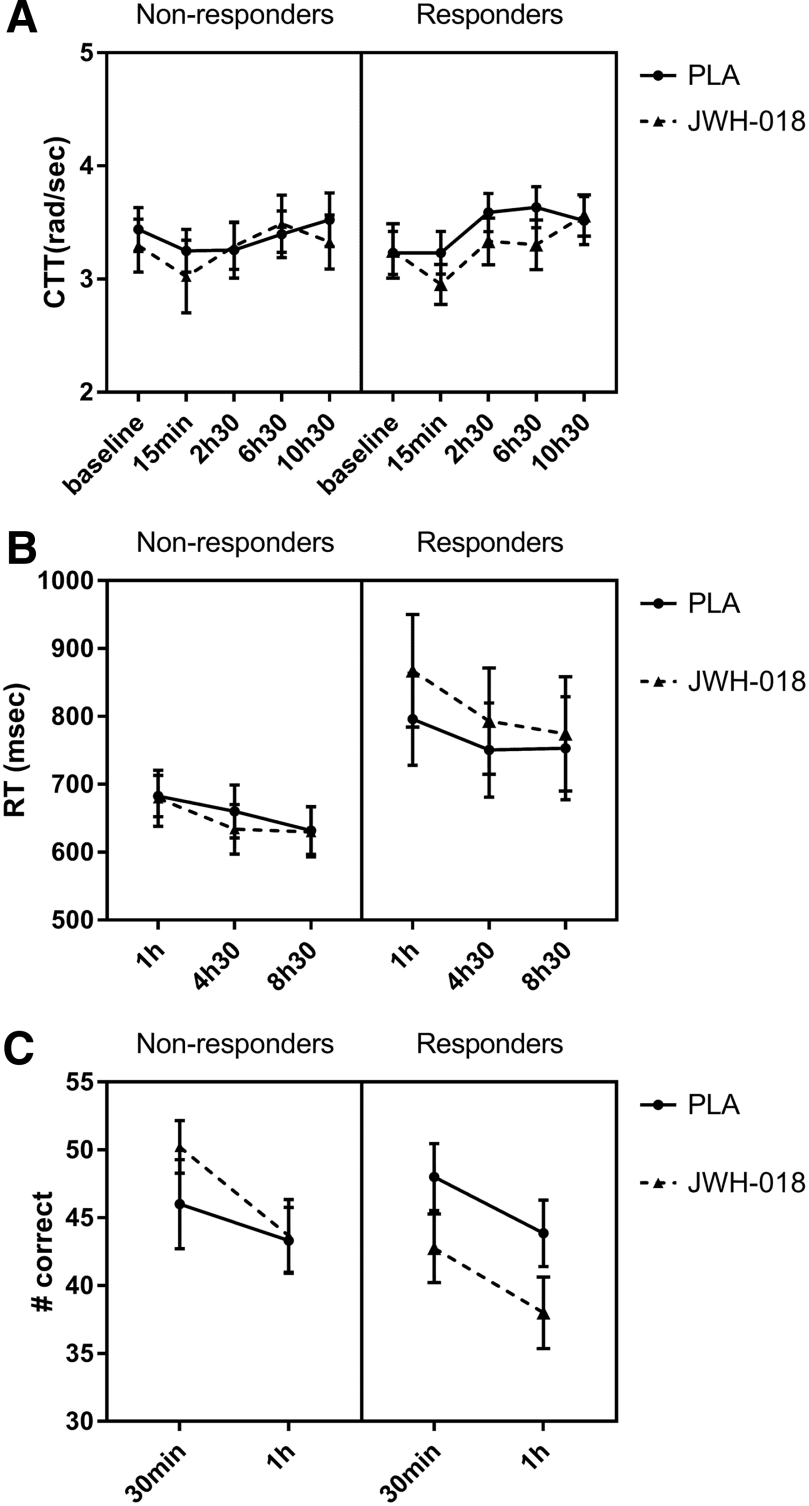
Mean (SEM) values for both groups for **(A)** lambda-c in the CTT, **(B)** RT on Go signals in the stop signal task, and **(C)** number of correct responses in the spatial memory task as a function of time after treatment with placebo (PLA) and JWH-018. CTT, critical tracking task; RT, reaction time.

Baseline critical tracking (CTT) scores did not show significant differences between treatments. CTT scores taken after administration showed a significant effect of Treatment (*F*_1,15_ = 5.52; *p*=0.017) and Time (*F*_3,45_=20.54; *p*<0.001) (Fig. 4), being lower for JWH-018 and increasing after administration.

In the stop signal test (SST), RTs on Go responses showed a significant effect of Time (*F*_1.4,20.9_=11.59; *p*<0.001) and a Treatment×Responder interaction (*F*_1,15_=3.73; *p*=0.036). RTs were slower in the responders under the influence of JWH-018, and decreased over time.

On the spatial memory test (SMT), a significant Treatment effect was found on the recall scores (*F*_1,14_=135,98; *p*<0.001), with decreased scores in the JWH-018 condition.

No significant effects were found for the performance scores of the DAT, TOL, and DSST.

### Subjective questionnaires

Results of the statistic tests are shown in Table 3. Individual and mean subjective high scores are shown in Figures 1 and 3. Highest subjective intoxication was reached at 1 h postadministration, with an average of 1.04 (±0.75) in the nonresponders and 6.35 (±2.9) in the responders. The effect of JWH-018 on subjective high followed a counter-clockwise hysteresis when plotted against serum concentrations over time, as shown in Figure 3.

**Table 3.** *F*-Value (Degrees of Freedom) and *p*-Value for the Significant Effects on the Different Scales of the Subjective Questionnaires

	Treatment		Time		Group		Treatment×time		Treatment×group		Treatment×time×group	
	*F*(df)	*p*	*F*(df)	*p*	*F*(df)	*p*	*F*(df)	*p*	*F*(df)	*p*	*F*(df)	*p*
Subjective high	18.79 (1,15)	<0.01	32.87 (11,165)	<0.01	17.65 (1,15)	<0.01	18.30 (11,165)	<0.01	18.57 (1,15)	<0.01	17.83 (11,165)	<0.01
POMS												
Anxiety			6.68 (2,24)	0.025				0.044				
Friendliness			5.02 (2,24)	0.007								
Confusion			16.32 (1.2,14)	<0.001			10.32 (2,24)	<0.001			7.44 (2,24)	0.002
Vigor	4.12 (1,12)	0.033										
Elation					3.31 (1,12)	0.047						
Depression									4.68 (1,12)	0.026		
Arousal							4.21 (2,24)	0.014	3.46 (1,12)	0.044		
Bowdle												
External perception	5.08 (1,15)	0.02	13.67 (1.3,19.6)	<0.001	4.76 (1,15)	0.023	9.8 (1.2,18.7)	0.002	3.3 (1,15)	0.045	5.7 (2,30)	0.004
Internal perception	3.29 (1,15)	0.045	6.31 (1.04,15.6)	0.011	3.65 (1,15)	0.038	6.1 (1.03,15.5)	0.013			4.59 (2,30)	0.009
High	20.27 (1,15)	<0.001	32.78 (1.06,15.8)	<0.001	22.1 (1,15)	<0.001	21.4 (1.05,15.7)	<0.001	20.55 (1,15)	<0.001	17.91 (2,30)	<0.001
Drowsiness			6.09 (1.2,17.6)	0.010	7.2 (1,15)	0.009	2.94 (1.15,17.3)	0.05	4.06 (1,15)	0.031	4.44 (2,30)	0.010
SCRQ												
Drug liking	4.93 (1,11)	0.024	7.19 (1,11)	0.011			6.77 (1,11)	0.013	14.12 (1,11)	0.002	9.55 (1,11)	0.005
CADSS												
Amnesia	3.63 (1,14)	0.039	4.2 (1.5,17)	0.025			4.2 (1.2,17)	0.025			2.6 (2,28)	0.046
Depersonalization	10.8 (1,14)	0.003	14.39 (1.03,14.4)	0.001	3.63 (1,14)	0.039	12.41 (1.03,14.5)	0.02	6.39 (1,14)	0.012	6.8 (2,28)	0.002
Derealization	4.85 (1,14)	0.023	10.0 (1.03,14.4)	0.003			5.45 (1.03,14.4)	0.017	3.64 (1,14)	0.039	4.58 (2,28)	0.01
Total score	5.64 (1,14)	0.016	10.75 (1.03,14.4)	0.003			6.32 (1.03,14.4)	0.011	4.02 (1,14)	0.033	4.99 (2,28)	0.007

**FIG. 5.**
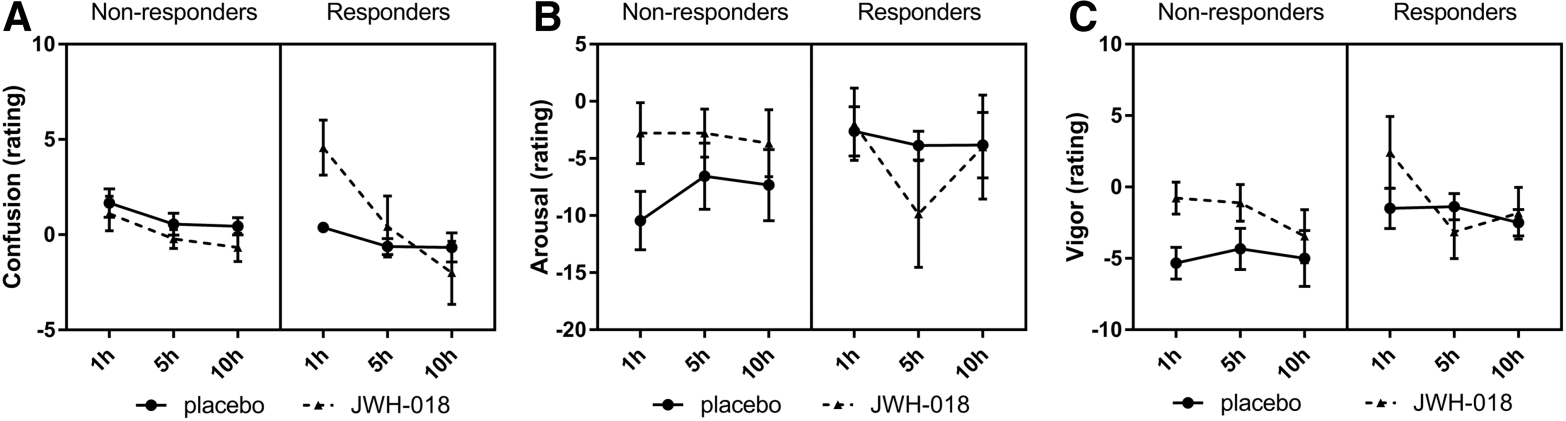
Mean (SEM) scores for the two groups for the Profile of Mood States scale's Confusion **(A)**, Arousal **(B)**, and Vigor **(C)** measured at different times after treatment.

POMS was taken at baseline and repeated three times after drug administration. Baseline scores differed between treatments; therefore, a baseline correction was performed (baseline score was subtracted from each sore at the subsequent time points). Scores for confusion were increased for the responders in the JWH-018 condition and decreased over time, while the scores were low and relatively constant in the placebo condition. Placebo resulted in lower scores on vigor. Responders scored higher on elation*,* while they scored lower on depression and arousal in the JWH-018 condition. Nonresponders' arousal scores improved under influence of JWH-018, while responders showed a slightly decreased arousal after JWH-018 (Fig. 5). No significant effects were found on the other mood states or scale of the POMS.

**FIG. 6.**
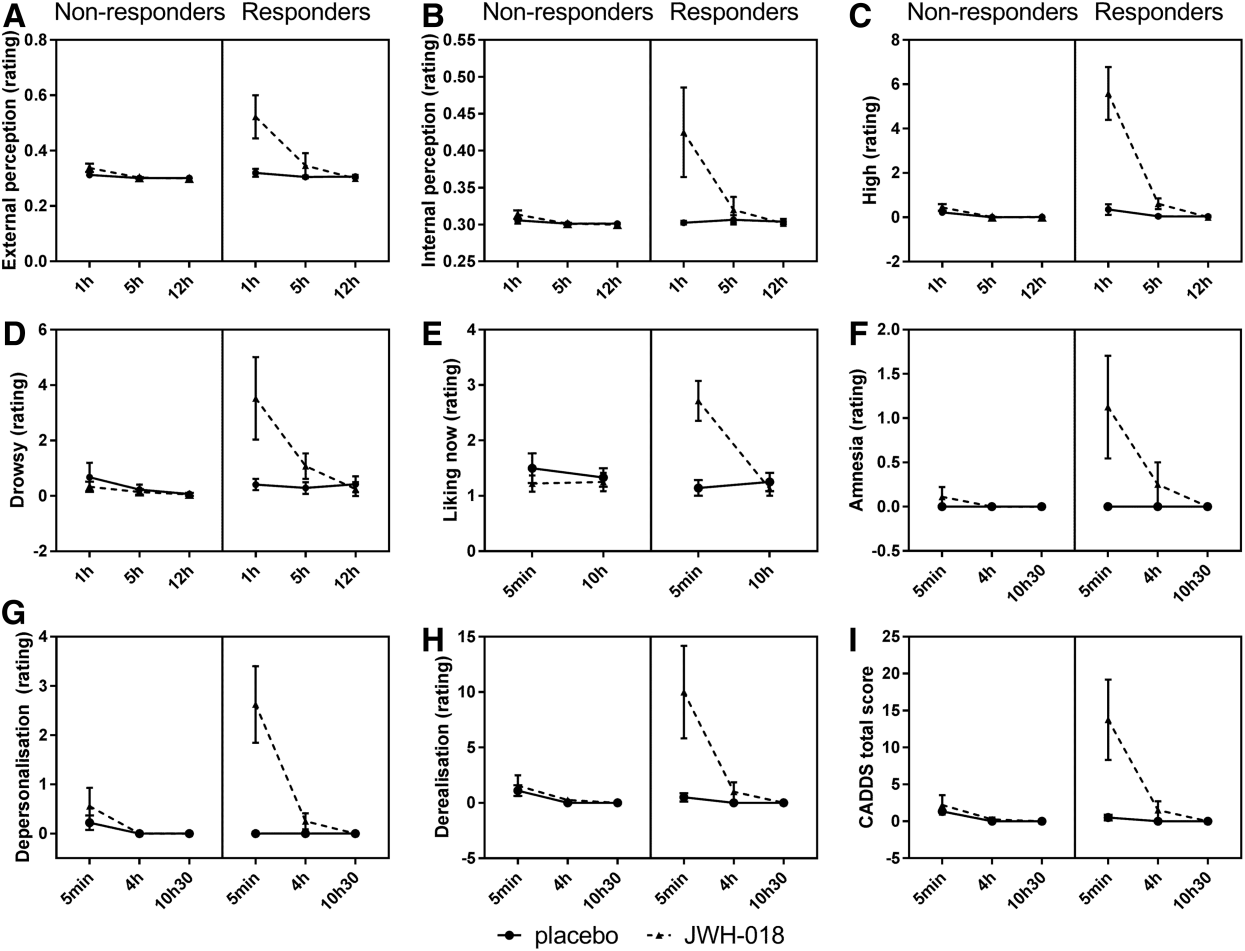
Mean (SEM) scores for the two groups for the Bowdle scale's External **(A)**, Internal **(B)**, High **(C)**, Drowsy **(D)**, the Sensitivity to Cannabis Reinforcement Questionnaire scale's Drug Liking now **(E)**, and the Clinician-Administered Dissociative States Scale's Amnesia **(F)**, Depersonalization **(G)**, Derealization **(H)**, and Total Score **(I)** measured at different times after treatment.

For the Bowdle visual analog scales, external perception ratings were higher for the responder group and after JWH-018, and scores decreased over time mainly due to a decrease in the JWH-018 condition. On the internal perception scale, JWH-018 induced higher scores at 1h after administration. On the high scale, JWH-018 caused higher scores especially at 1 h post-treatment in the responder group. Responders also showed more drowsiness after JWH-018 treatment (Fig. 6).

JWH-018 induced increased scores of drug liking of the SCRQ, especially in the responder group, which decreased over time (Fig. 6).

All scales of the CADSS showed higher scores after JWH-018 treatment, which decreased over time and all, except amnesia, demonstrated higher scores for the responders (Fig. 6).

No significant effects were found on the scales of the MCQ.

## Discussion

In a prior pilot study,^29^ we demonstrated that 2 and 3 mg of JWH-018 was well tolerated by recreational cannabis users (*N*=6) while producing some impairment in cognitive functioning.^29^ The present study is an expansion of that study, as we increased our sample size to *N*=17 and increased the dose of JWH-018 in these additional participants. The results showed that there was large variability in the subjective response to the drug, with some people reporting no subjective intoxication, while others reported maximal subjective intoxication. We therefore applied a median split on the subjective high score, and the factor responder (subjective high score >2 vs. subjective high score <2) was used as a between-subjects factor.

Besides the difference in subjective response to JWH-018, there were also clear differences in the serum concentrations of JWH-018 (max. 4.67 ng/mL in nonresponders; 10.59 ng/mL in responders). This variability in concentration, and consequently the variability in response, is believed to be partly due to variations in drug delivery. The JWH-018 and Knaster mixture was heated in glass pipes for about 15 sec, after which the vapor was inhaled in one take, implicating that participants only had one chance to inhale it correctly. Toxicological analyses of the glass pipes previously demonstrated that a substantial proportion (sometimes up to 70%) of the doses was not inhaled.^29,31^ For the participants who were added after the pilot, analyses showed residues in the pipes up to 83%. This again differed between the responders (average of 46% residue) and nonresponders (68%) and once more demonstrates the difficulty of controlling JWH-018 administration. Variations in drug delivery are likely to also impact levels of impairment and side effects when using synthetic cannabinoids in real-life settings. Users most often do not have any information on the type or dose of the synthetic cannabinoids in herbal mixtures sold on the street. In addition, the amount of active ingredient in herbal mixtures is not homogeneous within a brand and even within a package.^32^ The present study shows that inhalation of even small doses as low as 2 mg can unpredictably induce psychological effects that vary from weak to moderate. Successive inhalations from a given mixture may therefore provoke sudden and unexpected levels of impairments and increase the risk of overdosing.

Cases of acute intoxication due to the use of synthetic cannabinoids are commonly reported by emergency departments.^24^ In fatal cases, JWH-018 concentrations as high as 199 ng/mL (in whole blood) have been found.^33^ Lower concentrations of JWH-018, ranging from <0.10 to 13 ng/mL in serum, are reported in users with serious nonfatal side effects.^34^ The average concentration of 7.49 ng/mL JWH-018 (max 22.26 ng/mL) found in the present study falls within this latter range. However, this was determined 5 min after intake and declined rapidly over time (average of 2.07 and 1.44 ng/mL at 1 and 2 h postadministration). It is therefore to be expected that the acute concentrations of JWH-018 in case reports, where users are admitted to hospital a couple of hours after drug intake, were a lot higher than the concentrations that we have shown here. The present study also demonstrates a counter-clockwise hysteresis loop between drug concentration and subjective intoxication, indicating that although serum concentrations of JWH-018 reach their peak within 5 min, the peak in subjective intoxication is reached later. This implicates that there is a distribution delay between the systemic drug concentration and the time to reach the site of action, a phenomenon that is also known to occur after THC administration.^35^ The time difference between the peak in concentration and the peak in subjective intoxication further provides support for the median split based on subjective high instead of drug concentration.

A VAS rating of subjective high was used to distinguish participants who did not experience drug effects from those who did report subjective intoxication. Subjective high is a reliable estimate of the magnitude of drug intoxication and has been used in many studies involved with cannabis administration.^4,36–38^ In our study, ratings of subjective high in addition served as a control measure of actual drug delivery, which we expected to vary quite a lot between participants. Individual differences in dose inhalation, absorption, and metabolism were expected to impact on the subjective high experience and consequently on the level of performance impairment as assessed in objective tests, as well as feelings of mood and dissociation as assessed with questionnaires. This expectation was largely confirmed by the present data as high JWH-018 concentrations, performance impairments, and subjective experiences of amnesia, confusion, derealization and depersonalization were found in participants who experienced a subjective high (responders) compared with those who did not (nonresponders). This does not imply that nonresponders are insensitive to the influence of JWH-018, but indicates that JWH-018 concentrations in nonresponders were too low to generate a significant change in behavior.

The present study demonstrated that JWH-018 impaired tracking performance, RT, and memory, especially in the responder group. The other cognitive measures, which have previously been shown to be sensitive to the effects of cannabis,^4,8,13,39^ were not impaired by JWH-018. This implies that with the present dose and administration, the impairing effects of JWH-018 are less than that of cannabis. Therefore, a higher dose of JWH-018 or better administration procedure would be needed to achieve a behavioral impairment profile that is similar to a typical cannabis dose.

JWH-018 also affected subjective measures; primarily in the responder group, JWH-018 induced dissociative effects such as changes in internal and external perception, which were previously also demonstrated after THC administration.^40^ Additional dissociative symptoms such as feelings of amnesia, derealization, and depersonalization were also increased during JWH-018 intoxication, similar to that found in cannabis intoxication.^41^ Subjective intoxication and feelings of confusion were also higher in the responders compared with nonresponders. These subjective symptoms of dissociation, amnesia, and confusion are prominently reported in cases of synthetic cannabinoid overdosing, and the resulting behavioral pattern is often referred to as a zombie effect.^21,42,43^ In the present study, low doses of JWH-018 appeared to result in the first signs of such zombie symptoms.

The present study was the first controlled experimental study in a sufficiently large sample assessing the physiological, subjective, and behavioral effects of JWH-018. The relatively low dose of JWH-018 impaired cognitive performance and induced subjective effects, and also showed a large variation in subjective intoxication. It is reasonable to assume that the serious side effects often seen in overdose cases are due to higher doses and/or combinations of different synthetic cannabinoids in smoking mixtures.

## Supplementary Material

Supplemental data

## Abbreviations Used

BMI: body mass index
CADSS: Clinician-Administered Dissociative States Scale
CTT: critical tracking task
DAT: divided attention task
DSMB: Data Safety Monitoring Board
DSST: digit symbol substitution task
ECG: electrocardiogram
MCQ: Marijuana Craving Questionnaire
NPS: novel psychoactive substances
POMS: Profile of Mood States
RT: reaction time
SCRQ: Sensitivity to Cannabis Reinforcement Questionnaire
SD: standard deviation
SEM: standard error of the mean
SMT: spatial memory task
SpO2: saturation of peripheral oxygen
SST: stop signal task
THC: Δ9-tetrahydrocannabinol
TOL: Tower of London
VAS: visual analog scale
VAS-high: visual analog scales of subjective high
