# Supplementary material for: Neurocognition and Subjective Experience Following Acute Doses of the Synthetic Cannabinoid JWH-018: Responders Versus Nonresponders
Source: Cannabis Cannabinoid Res. 2019 Mar 13;4(1):51–61. doi: 10.1089/can.2018.0047 (PMC6661919; doi:10.1089/can.2018.0047)
Supplement: Supplemental data [file Supp_Data.pdf]

Supplementary Data

Procedures

Before the first test day, subjects were trained extensively in all cognitive tests to minimize practice effects. Subjects were asked to refrain from using alcohol or caffeine on the test day or the day before testing. Smoking was prohibited for 30 min before and during test days. Subjects were asked to arrive at the site well rested. On each test day, subjects were instructed to have a standard breakfast before coming to the site. They received lunch and dinner at the site. Subjects were instructed to continue their cannabis use as normal but were requested to abstain from cannabis for about 5 days before the test day, to make sure they were negative on the test day.

Test days took place at the testing facilities at Maastricht University. Participants spent the day in a test room equipped with a bed, chair, and table with laptop. The test room was adjacent to a bathroom and a room where the researchers were seated. An alcohol breath test and drug urine tests were performed on arrival to assess the presence of alcohol, morphine, cocaine, cannabis, methamphetamine, and amphetamine. Subsequently, an intravenous catheter was placed in the lower arm. Urine and blood samples were taken at baseline and at the end of the test day to perform laboratory analyses (hematology and blood chemistry, urinalysis). Blood samples were taken at regular intervals during the test days to determine pharmacokinetics. Blood pressure and heart rate were measured at regular intervals (every 10 min within the first hour, every 15 min between 1 and 2 h, and every 30 min between 2 and 12 h after inhalation) during the test day using an Omron upper arm blood pressure monitor (Model M6 AC; Omron Healthcare Europe). Cognitive tests and subjective questionnaires were taken with regular intervals up until 12 h after administration (Tables 1 and 2). In between test batteries, subjects could repose at the site, where they could watch TV, read, or use the Internet. Test days always started at the same time in the morning.

Safety

A Dyna-Vision ambulatory patient monitoring system (model DVM-012SG-LCD; Techmedic International, The Netherlands) was used to continuously measure and transmit electrocardiogram (ECG) and vital signs in real time to the medical supervisor. The signal was transmitted to the laptop as well as the smartphone of the medical supervisor. The patch with sensors was attached to the subject's chest and connected to a receiver carried by the participant around his/her neck or middle. The system transmits three lead ECGs, saturation of peripheral oxygen (SpO2), plethysmogram, respiration and skin temperature, and beat-to-beat noninvasive blood pressure. Alert signals were given every time one of the measures was above or below the normal range. The medical doctor would determine whether the value was clinically significant or not. The medical supervisor was present at the facility during the whole test day and could check on the participant whenever he/she experienced any complaints, or when any of the ECG or vital sign measures indicated a value outside the normal range.

Blood and urine samples were taken at baseline and at the end of the test day for laboratory analyses. Hematology (e.g., total leukocytes), clinical chemistry (e.g., renal and liver function tests), and urinalysis (e.g., kidney and urinary tract functioning tests) were performed. Reference ranges were used to determine whether laboratory values were within the normal range. In case a measured parameter was outside the normal ranges, the medical supervisor decided whether or not it was considered a clinically significant deviation. In this decision, the other parameters, the overall condition of the participant, and the baseline values were taken into account. At the end of the test day, participants were given a diary on which they were asked to take note of any possible side effect that they experienced up until 72 h after administration of the drug.

An independent Data Safety Monitoring Board (DSMB) was installed to inspect and evaluate all vital, safety, and behavioral data collected throughout the study. Data were submitted to the DSMB halfway and at the end of every active dose condition. The study was continued only after a positive evaluation from the DSMB.

Performance tests

Digit symbol substitution task

The digit symbol substitution task is a computerized version of the original paper and pencil test taken from the Wechsler Adult Intelligence Scale.[S1] The participant is required to match each digit with a symbol from the encoding list as rapidly as possible. The number

of digits correctly encoded within 3 min is the performance measure.

Critical tracking test

The critical tracking task measures the subject's ability to control a displayed error signal in a first-order compensatory tracking task.[S2] Error is displayed as a horizontal deviation of a cursor from the midpoint on a horizontal, linear scale. Compensatory joystick movements null the error by returning the cursor to the midpoint. The frequency at which the subject loses control is the critical frequency or λ_c. The test included five trials of which the lowest and the highest scores were removed; the average of the remaining scores is taken as the final score.

Divided attention task

The divided attention task measures the ability to divide attention between two tasks performed simultaneously.[S3] Subjects have to perform the same tracking task as described above but now at a constant level of difficulty. As a secondary task, the subject monitors 24 single digits presented in the corners of the computer screen. The subjects are instructed to react to the target number "2" by removing their foot as fast as possible from a pedal switch. Mean absolute tracking error (in mm) and number of control losses are the performance measures of the primary task. Number of correct responses and mean reaction time (msec) of the responses to the target number are the performance measures in the secondary subtask.

Stop signal task

The stop signal task measures motor impulsivity, which is defined as the inability to inhibit an activated or precued response leading to errors of commission. The present test is adapted from an earlier version[S4] and has been validated for showing stimulant and sedative drug effects.[S5] The task requires subjects to make quick responses to visual go signals and to inhibit their response if a subsequent visual stop signal, that is, "*," appeared in one of the four corners of the screen. Dependent variables are go reaction time, stop reaction time, response accuracy, and omission (not responding on go-trials) and commission errors (not inhibiting a no go-trial). Stop reaction time represents the estimated mean time required to inhibit a response. Stop reaction time is calculated by subtracting the stop signal delay from the reaction time on go-trials associated with n-th percentile of the reaction time distribution.[S6]

Tower of London

The Tower of London is a decision-making task that measures executive function and planning.[S7,S8] The task consists of computer-generated images of begin- and end-arrangements of three colored balls on three sticks. The subject's task is to determine as quickly as possible whether the end-arrangement can be accomplished by "moving" the balls in two to five steps from the beginning arrangement by pushing the corresponding number-coded button. The total number of correct decisions is the main performance measure.

Spatial memory task

Ten black-and-white pictures are presented subsequently in 10 different locations on a computer screen. After presentation, each picture is presented alone with two possible locations where it appeared. Participants' task is to choose the correct location; a measure of immediate recall phase. This procedure is repeated six times with different stimuli and locations. After a 30-min delay, the recall phase is repeated; this test serves as a delayed recall measure (adapted from Kessels et al.[S9]).

Subjective Questionnaires

Profile of Mood States

The Profile of Mood States, POMS, is a self-assessment mood questionnaire with 72 items, rated on a 5-point Likert scale, with 0 being "not at all" to 4 "extremely." Subjects have to indicate to what extent these items were representative of their mood at that moment in time. Eight mood states are classified and quantified by calculating the sum score of associated items for each mood state, that is, anxiety, depression, anger, vigor, fatigue, confusion, friendliness, and elation. Two composite scales are derived: arousal and positive mood.[S10]

Bowdle visual analog scales

Psychedelic effects are assessed using a 13-item visual analog scale.[S11] Two scales measure subjective "high" and "drowsiness." From the other scales, composite scores of "internal perception" and "external perception" are calculated.

The Marijuana Craving Questionnaire

The Marijuana Craving Questionnaire is a 12-item self-report instrument that assesses cannabis craving/wanting along four dimensions: (1) compulsivity, an inability to control cannabis use; (2) emotionality, use of cannabis in anticipation of relief from withdrawal or negative

mood; (3) expectancy, anticipation of positive outcomes from smoking cannabis; and (4) purposefulness, intention, and planning to use cannabis for positive outcomes. Items are scored on a 7-point scale ranging from strongly disagree to strongly agree.[S12]

Sensitivity to Cannabis Reinforcement Questionnaire

This questionnaire asks subjects to rate their liking and wanting of cannabis use during their present condition and in general. Subjects are asked four questions: How pleasant is using cannabis right now (drug liking), how much do you want to use cannabis right now (drug wanting), how pleasant is using cannabis in general, and how much do you want to use cannabis in general? Subjective valence of liking and wanting is scored on a 5-point scale: 1 = somewhat, 2 = slightly, 3 = moderately, 4 = very, and 5 = extremely.

Clinician-Administered Dissociative States Scale

The Clinician-Administered Dissociative States Scale (CADSS)[S13] comprises 19 subjective items, ranging from 0 "not at all" to 4 "extremely." It is divided into three components: (1) depersonalization, (2) derealization, and (3) amnesia.[S14] Summed together, these subscales form a total dissociative score. The CADSS is specifically designed to be a standardized measure of present-state dissociative symptomatology.

Pharmacokinetics. Fourteen blood samples (5 mL) were taken during each test day (i.e., at baseline and 5, 15, 30, 45 min, and 1, 1.5, 2, 3, 4, 6, 8, 10, and 12 h after inhalation). These were centrifuged and serum was frozen at –20°C until pharmacokinetic assessments.

The glass pipes used for the administration of the 75 μg/kg body weight dose were analyzed for residual amounts of JWH-018 (see Toennes et al.[S15] for a description of the methods).

Supplementary References

S1. McLeod DR, Griffiths RR, Bigelow GE, et al. An automated version of the digit symbol substitution test (DSST). Behav Res Methods. 1982;14:463–466.

S2. Jex HR, McDonnell JD, Phatak AV. A "critical" tracking task for man-machine research related to the operator's effective delay time. I. Theory and experiments with a first- order divergent controlled element. NASA CR-616. NASA Contract Rep NASA CR. 1966:1–105.

S3. Moskowitz H. Laboratory studies of the effects of alcohol on some variables related to driving. J Safety Res. 1973;5:185–192.

S4. Fillmore MT, Rush CR, HaysL. Acute effects of oral cocaine on inhibitory control of behavior in humans. Drug Alcohol Depend. 2002;67:157–167.

S5. Ramaekers JG, Kuypers KPC. Acute effects of 3,4-methylenedioxymethamphetamine (MDMA) on behavioral measures of impulsivity: alone and in combination with alcohol. Neuropsychopharmacology. 2006;31:1048–1055.

S6. Logan GD. On the ability to inhibit thought and action. In: Inhibitory processes in attention, memory and language, Dagenbach D and Carr TH, eds. Academic Press: San Diego, CA, pp. 189–239, 1994.

S7. Shallice T. Specific impairments of planning. Philos Trans R Soc Lond B Biol Sci. 1982;98:199–209.

S8. Sobczak S, Riedel W, Booij I, et al. Cognition following acute tryptophan depletion: difference between first-degree relatives of bipolar disorder patients and matched healthy control volunteers. Psychol Med. 2002;32: 503–515.

S9. Kessels RP, Postma A, de Haan EH. Object relocation: a program for setting up, running, and analyzing experiments on memory for object locations. Behav Res Methods. 1999;31:423–428.

S10. de Wit H, Enggasser JL, Richards JB. Acute administration of d-amphetamine decreases impulsivity in healthy volunteers. Neuropsychopharmacology. 2002;27:813–825.

S11. Bowdle AT, Radant AD, Cowley DS, et al. Psychedelic effects of ketamine in healthy volunteers: relationship to steady-state plasma concentrations. Anesthesiology. 1998;88:82–88.

S12. Heishman SJ, Singleton EG. Assessment of cannabis craving using the Marijuana Craving Questionnaire. Methods Mol Med. 2006;123:209–216.

S13. Bremner JD, Krystal JH, Putnam FW, et al. Measurement of dissociative states with the clinician-administered dissociative states scale (CADSS). J Trauma Stress. 1998;11:125–136.

S14. Zuurman L, Roy C, Schoemaker RC, et al. Effect of intrapulmonary tetrahydrocannabinol administration in humans. J Psychopharmacol. 2008; 22:707–716.

S15. Toennes SW, Geraths A, Pogoda W, et al. Pharmacokinetic properties of the synthetic cannabinoid JWH-018 and of its metabolites in serum after inhalation. J Pharm Biomed Anal. 2017;140:215–222.
